# Supplementary material for: A meta-analysis of threats to valid clinical inference in preclinical research of sunitinib
Source: eLife. 2015 Oct 13;4:e08351. doi: 10.7554/eLife.08351 (PMC4600817; doi:10.7554/eLife.08351)
Supplement: Figure 1—source data 1. — DOI: http://dx.doi.org/10.7554/eLife.08351.004 [file elife08351s001.docx]

**A**

| **Internal Validity Characteristics** | **Coding Parameters** |
| --- | --- |
| **Exact sample size given for all groups** | Exact sample sizes are those reported as a distinct number of animals per group, as opposed to a range or absence of sample size information. In the case of a range, the mean number of animals was used in meta-analysis. |
| **Performance of sample size calculation** | Whether or not a sample size calculation was performed for the experiment in question, either *a priori* or after the experiment’s completion. |
| **Randomly allocated to treatment** | If the experiment was said to have assigned treatments in a random manner, irrespective as to whether the method of randomization was specified. |
| **Concealed allocation** | If the experiment was said to have concealed the treatment allocation from personnel before or during the experiment, irrespective as to whether the method of concealment was specified. |
| **Blinded outcome assessment** | If the experiment was said to have blinded outcome assessors from treatment allocation during the experiment, irrespective as to whether the method of blinding was specified. |
| **Used named inferential statistical test** | If the experiment reported the results of an inferential statistical test (e.g. a p-value), reported details of statistical test methods were recorded. |
| **Addressed animal flow through experiment** | Any details regarding the attrition of experimental animals. These include a) declaration that no attrition throughout experiment, b) attrition of animals after first administration, but before outcome assessment, c) attrition of animals during outcome assessment, d) attrition of animals during analysis (e.g. outliers) or e) non-specified attrition (e.g. some animals lost, but not clear when lost). We defined "attrition" to include animals removed from the experiment due to toxicity, toxic death, or a reason unrelated to treatment (e.g. illness in animal). |
| **Evaluated dose-response (≥3 doses)** | Whether the experiment describes a dose response relationship (tests disease response at ≥ 3 non-zero doses of sunitinib)? The number of doses was recorded for each experiment. |
| **Construct Validity Characteristics** |  |
| **Species** | The species of animal used in the experiment was recorded (e.g. mouse, rat) |
| **Age** | For mice: Pediatric/Juvenile= 0-8 weeks, Adult= 8-72 weeks, Aged= 72+ weeks For rats: Pediatric/Juvenile= 0-7 weeks Adult= 7+ weeks.[[52](#_ENREF_52)] |
| **Immune Status** | Whether or not the experimental animals possessed an intact immune system (immunocompetent) or were immunocompromised (e.g. Nude, SCID). |
| **Sex** | Whether the experimental animals were all male, all female, or mixed sex was recorded. |
| **Model Type** | Disease models that included tumour tissue transplantation (either cells or tissue) were classified as wither being xenografts (e.g. human) or allografts by assessing the animal strain and the provenance of the tumour material. Additional disease models included genetically engineered mouse models (GEMMs; e.g. MMTV-PyMT breast cancer model) or those that fell outside of the aforementioned categories (e.g. other models such as carcinogen-induced tumour models). |
| **Experiment type** | For tumour transplantation models, first drug administration at <200 mg/mm^3^ is considered to be an early-stage disease model, and >200 mg/mm3 is considered advanced-stage[[53](#_ENREF_53), [54](#_ENREF_54)]. We assumed the same tumour size standards for rats as for mice. |
| **Evidence for causal mechanism** | The presence of other types of experiments showing treatment effect (other than our primary outcome measure of tumour volume) was recorded at multiple steps in the causal pathway of the disease mechanism. Note: Classification pertains to the purpose behind the experiment rather than just the experimental technique used to investigate.  Molecular: All experiments measuring changes to molecules within the confines of a single cell. Included in this are mechanisms of action (e.g. proximal molecular events from receptors to second messengers to transcription factors) and expression levels of important proteins or nucleic acids.  Physiological: All experiments measuring changes that affect or occur at the level of groups of cells, tissues, organs, etc. These changes include single molecular messengers such as secreted chemokines, the proportion of cells in a tissue undergoing a cellular process such as apoptosis, mitosis, or hypoxia, and more macro measurements such as the blood perfusion of a tissue/tumour or microvessel density.  Functional/Clinical/Behavioural: All experiments measuring changes at the level of the entire organism (e.g. behavioural testing) |

**Figure 1 – source data 1A: Coding details for IV and CV categories.**
