# Supplementary material for: A meta-analysis of threats to valid clinical inference in preclinical research of sunitinib
Source: eLife. 2015 Oct 13;4:e08351. doi: 10.7554/eLife.08351 (PMC4600817; doi:10.7554/eLife.08351)
Supplement: Table 1—source data 1. — (C) Search Strategies. (D) PRISMA Flow Diagram. (E) Demographics of included studies at qualitative level. DOI: http://dx.doi.org/10.7554/eLife.08351.007 [file elife08351s002.docx]

**Table 1 – source data 1C: Search Strategies**

*MEDLINE Search Strategy*

Database: Ovid MEDLINE(R) In-Process & Other Non-Indexed Citations and Ovid MEDLINE(R) <1946 to Present>

Search Strategy:

--------------------------------------------------------------------------------

1 exp animal experimentation/ or exp models, animal/ or animals/ or mammals/ or vertebrates/ or exp fishes/ or exp amphibia/ or exp reptiles/ or exp birds/ or exp hyraxes/ or exp marsupialia/ or exp monotremata/ or exp scandentia/ or exp chiroptera/ or exp carnivora/ or exp cetacea/ or exp Xenarthra/ or exp elephants/ or exp insectivora/ or exp lagomorpha/ or exp rodentia/ or exp sirenia/ or exp Perissodactyla/ or primates/ or exp strepsirhini/ or haplorhini/ or exp tarsii/ or exp platyrrhini/ or catarrhini/ or exp cercopithecidae/ or gorilla gorilla/ or pan paniscus/ or pan troglodytes/ or exp pongo/ or exp hylobatidae/ or hominidae/

2 Drug Evaluation, Preclinical/

3 ((animal$1 or chordata or vertebrate* or fish$2 or amphibian* or amphibium* or reptile$1 or bird$1 or mammal* or dog or dogs or canine$1 or cat or cats or hyrax* or marsupial* or monotrem* or scandentia or bat or bats or carnivor* or cetacea or edentata* or elephant* or insect or insects or insectivore or lagomorph* or rodent$2 or mouse or mice or murine or murinae or muridae or rat or rats or pig or pigs or piglet$1 or swine or rabbit$1 or sheep$1 or goat$1 or horse$1 or equus or cow or cows or cattle or calf or calves or bovine or sirenia or ungulate$1 or primate$1 or prosimian* or haplorhini* or tarsiiform* or simian*or platyrrhini or catarrhini or cercopithecidae or ape or apes or hylobatidae or hominid* or chimpanzee* or gorilla* or orangutan* or monkey or monkeys or ape or apes) adj5 (trial$1 or study or studies or experiment* or laborator$3 or research*)).tw.

4 (preclinical* or pre-clinical*).tw.

5 or/1-4

6 (Sunitinib or Sutent).tw.

7 ("SU 011248" or "SU 11248" or "SU-011248" or "SU-11248" or SU011248 or SU11248 or "su 010398" or "su 10398" or su010398 or su10398).tw.

8 ("UNII-V99T50803M" or "HSDB 7932" or "pha 2909040ad" or pha2909040ad).tw.

9 or/6-8

10 5 and 9

*Embase Search Strategy*

Database: Embase Classic+Embase <1947 to 2012 February 22>

Search Strategy:

--------------------------------------------------------------------------------

1 exp animal experiment/ or exp animal model/ or animal/ or exp invertebrate Chordata/ or exp experimental animal/ or exp transgenic animal/ or exp male animal/ or exp female animal/ or exp juvenile animal/ or vertebrate/ or exp fish/ or exp amphibia/ or exp reptile/ or exp bird/ or mammal/ or exp hyrax/ or exp marsupial/ or exp monotremate/ or exp scandentia/ or placental mammals/ or exp bat/ or exp carnivora/ or exp cetacea/ or exp edentata/ or exp elephant/ or exp insectivora/ or exp lagomorph/ or exp rodent/ or exp sirenia/ or exp ungulate/ or primate/ or exp prosimian/ or haplorhini/ or exp tarsiiform/ or simian/ or exp platyrrhini/ or catarrhini/ or exp cercopithecidae/ or ape/ or exp hylobatidae/ or hominid/ or exp chimpanzee/ or exp gorilla/ or exp orang utan/ (5157845)

2 ((animal$1 or chordata or vertebrate* or fish$2 or amphibian* or amphibium* or reptile$1 or bird$1 or mammal* or dog or dogs or canine$1 or cat or cats or hyrax* or marsupial* or monotrem* or scandentia or bat or bats or carnivor* or cetacea or edentata* or elephant* or insect or insects or insectivore or lagomorph* or rodent$2 or mouse or mice or murine or murinae or muridae or rat or rats or pig or pigs or piglet$1 or swine or rabbit$1 or sheep$1 or goat$1 or horse$1 or equus or cow or cows or cattle or calf or calves or bovine or sirenia or ungulate$1 or primate$1 or prosimian* or haplorhini* or tarsiiform* or simian*or platyrrhini or catarrhini or cercopithecidae or ape or apes or hylobatidae or hominid* or chimpanzee* or gorilla* or orangutan* or monkey or monkeys or ape or apes) adj5 (trial$1 or study or studies or experiment* or laborator$3 or research*)).tw.

3 (preclinical* or pre-clinical*).tw.

4 or/1-3

5 exp sunitinib/

6 (Sunitinib or Sutent).tw.

7 ("SU 011248" or "SU 11248" or "SU-011248" or "SU-11248" or SU011248 or SU11248 or "su 010398" or "su 10398" or su010398 or su10398).tw.

8 ("UNII-V99T50803M" or "HSDB 7932" or "pha 2909040ad" or pha2909040ad).tw.

9 557795 19 4.rn.

10 or/5-9

11 4 and 10

**D**

**Table 1 – source data 1D: PRISMA Flow Diagram**

**E**

*Does not add to 100% as many studies declared more than one funding source.

**Table 1 – source data 1E: Demographics of included studies at qualitative level**
