# Supplementary material for: A meta-analysis of threats to valid clinical inference in preclinical research of sunitinib
Source: eLife. 2015 Oct 13;4:e08351. doi: 10.7554/eLife.08351 (PMC4600817; doi:10.7554/eLife.08351)
Supplement: Figure 2—source data 1. — DOI: http://dx.doi.org/10.7554/eLife.08351.009 [file elife08351s003.docx]

**B**

| **Malignancy Sub-group** | **I^2^** |
| --- | --- |
| Acute Myelogenous Leukemia | 94% |
| Bladder Cancer | N/A |
| Breast Cancer | 69% |
| Colorectal Cancer | 75% |
| Gastrointerstinal Stromal Tumour | 87% |
| Hepatocellular Carcinoma | N/A |
| High-Grade Glioma | 10% |
| Melanoma | 85% |
| Multiple Myeloma | 45% |
| Neuroblastoma (Pediatric) | N/A |
| Non-Hodgkin Lymphoma | 0% |
| Non-Small-Cell Lung Carcinoma | 0% |
| Ovarian Cancer | 79% |
| Pancreatic Cancer | 89% |
| Prostate Cancer | 20% |
| Renal Cell Carcinoma | 85% |
| Small-Cell Lung Carcinoma | 93% |
| Soft Tissue Sarcoma | N/A |
| Squamous Cell Carcinoma of skin | N/A |
| Thyroid Cancer | N/A |
| Uncertain Malignancy | 69% |
| Overall | 83% |

Note: I^2^ listed as N/A for sub-groups with only one experiment (n=1)

**Figure 2 – source data 1B: Heterogeneity statistics (I^2^) for each malignancy sub-group.**
